# Adapting competence development to multicultural healthcare teams: a qualitative study of the International Caregiver Development Programme (ICDP) in nursing homes

**DOI:** 10.1186/s12912-026-04299-3

**Published:** 2026-01-09

**Authors:** Line Constance Holmsen, Bodil Tveit, Ane-Marthe Solheim Skar, Marit Helene Hem

**Affiliations:** 1 Centre of Diaconia and Professional Practice, VID Specialized University (VID), Oslo, Norway; 2 Faculty of Health Science, VID Specialized University, Oslo, Norway; 3 Division for Health Services, Norwegian Institute of Public Health, Oslo, Norway; 4 Faculty of Health Science, VID Specialized University, Oslo, Norway

**Keywords:** Health care workers, International Caregiver Development Programme (ICDP), Nursing homes, Person-Centred care, Competence development

## Abstract

**Background:**

Enhancing holistic, biopsychosocial and person-centred care for older persons depends on developing competence in psychosocial care. To decrease the theory-practice-gap in person-centred care, there is a need for research to investigate adaptions of competence development within person-centred care that enables knowledge integration and reflexivity to practice. More research is needed on competence development in person-centred care that is tailored to the nursing home context.

**Methods:**

This study aimed to explore how group leaders in International Caregiver Development Programme (ICDP) facilitated competence development within psychosocial care for multicultural healthcare teams in nursing homes. The qualitative design included five participatory observation sessions during the supervision of ICDP group leaders, one focus group interview conducted after the completion of ICDP, and the group leaders’ written logs and reflections from the ICDP group meetings. The data were analysed using hermeneutic analysis.

**Results:**

Three main themes were developed from the analysis:1) Creating the right atmosphere, consisting of (a) creating safety for openness, (b) highlighting mastery in practice and (c) helping ICDP participants to be mentally attuned; 2) Making the ICDP understandable, encompassing (a) transitioning to a reflective mode and (b) adapting the language level; and 3) Creating an inclusive and active learning environment, with (a) facilitating collective participation and (b) supporting the groups’ engagement as subthemes.

**Conclusions:**

Study findings suggest that interventions for psychosocial competence development require adjustments based on healthcare workers’ need for security, a sense of mastery, present-moment awareness, reflection on practice, appropriate language level, commitment and motivation. Such adaptations may be crucial for healthcare workers’ ability to integrate knowledge, reflexivity and sensitivity into person-centred practice. ICDP appears to be flexible and adaptable to a nursing context. Further research is needed on the ICDP in relation to professional confidence, sick leave and sustainability.

**Clinical trial number:**

Not applicable.

**Supplementary Information:**

The online version contains supplementary material available at https://doi.org/10.1186/s12912-026-04299-3.

## Background

This study focused on competence development within psychosocial and person-centred care (PCC) in nursing homes (NHs). Competence within PCC can be understood as the ability to support each resident as a unique person with various biomedical, psychosocial, spiritual and existential needs [1–3]. Rather than being considered as two separate concepts, psychosocial care - which focuses on emotional, social, and relational aspects - constitutes an essential dimension of successful, holistic person-centred practice. Healthcare workers’ (HCWs) competence in addressing older persons psychosocial needs is crucial for enhancing or maintaining the quality of life of NH residents and preventing functional decline [4–6]. Research shows that there is a theory-practice gap in PCC for older persons [7–11], due to a lack of a clear definition of PCC, limited knowledge of operationalization in practice [7], and insufficient resources in NHs. Health education and practice in NHs is described as dominated by medical knowledge [8] and task focused care [1, 10]. The integration of holistic, biopsychosocial care and PCC for older persons is dependent on further education and competence development within psychosocial care [2, 12, 13]. Studies highlight the need for more research on appropriate continuing education within PCC that is adapted to HCWs [9], as well as interventions that are tailored to the NH setting [14]. This study explores how competence development within psychosocial care is facilitated through the International Caregiver Development Programme (ICDP) for HCWs in NHs.

### Competence development within psychosocial and person-centred care

PCC is regarded as the gold standard in healthcare services for older persons [15], and, for over a decade, it has been recommended in guidelines for healthcare services for older persons [16, 17]. Kitwood’s [13] significant contribution to PCC was prompted by his observations regarding a dominance of the biomedical dimension in care of persons with dementia diagnosis. In line with Kitwood’s [13] descriptions of PCC, ICDP is a psychosocial approach to the development of care for older persons [2, 18].

Kitwood [13], Tashiro et al. [19] and Westerhof [20] describe how the transition to PCC requires an understanding of the relationship between HCWs and residents. These relationships develop through collaboration over time, with the residents’ individual needs at the centre. Westerhof [20] points out that there does not need to be a conflict between task-focused somatic care and relational PCC in NHs. Although practice is often dominated by task-oriented somatic care [21], many situations may still offer opportunities to incorporate PCC. Westerhof et al. [20] argue that, here, it is crucial that HCWs are able to shift between task-focused care and person-centred approaches as needed. Tasiro et al. [19] highlight how the complexity of HCWs’ responsibilities, along with increasing demands for contextually appropriate interaction, call for tools that support reflection on one’s practice as a part of competence development in PCC. Such reflections are often sparked by challenges in practice and involve describing specific situations in detail to help HCWs recognize emotions, thoughts, and personal reactions, as well as to critically analyse the interaction. In line with studies on counselling and ethical reflection groups [22–24], the ability to reflect on one’s practice promotes greater awareness and transparency regarding HCWs’ own skills [19]. Reflexivity in practice also supports continuous learning processes and helps to bridge the gap between theory and practice in PCC [19].

Røsvik [25] emphasizes that the HCWs’ sensitivity to NH residents’ needs is essential for the sustainability of PCC. In line with this, competence development in psychosocial care and PCC should strengthen HCWs ability to notice interpersonal communication [26] and interpret both interactions from intrapersonal and relational perspectives. This supports the potential for PCC, culturally sensitive care [13, 27, 28] and shared decision-making [29].

### Competence development that is tailored for the nursing home setting

Gabbay and LeMay [30] suggest that it is more likely the HCWs “mindlines”, than governmental guidelines that influence the development of practice. They argue that it is more fruitful to understand practice development as integration of new knowledge into HCWs’ experience of practice; called “mindlines”. Gabbay and LeMay’s (2004) define the term “mindlines” as internalized guidelines that is a blend of explicit evidence and personal and collective tacit knowledge that is shaped by experience and informal networks. The integration process and development of “mindlines” is complex and contextual, merging from reorientation and negotiation within a team working together [31]. Incorporating new knowledge into HCWs mindlines requires learning processes that are contextual, practice-oriented and team-based.

Research highlights the importance of tailoring interventions for HCWs to the NH context [14]. HCWs in NHs represent a diverse workforce in terms of education, experience, culture and language [32]. Studies show that HCWs in this setting often face challenges, such as poorly defined tasks and roles, insufficient competence and confidence, staff shortages, a lack of resources, limited proficiency in the native language and issues related to diversity and discrimination [32–37].

Competence development within the NH context may be affected by HCWs’ engagement, their relationships with intervention facilitators, expectations around roles, time constraints, leadership involvement and inadequate management [14, 38]. Manson et al. [38] recommend face-to-face learning delivered through simple, flexible and individualised interventions to ensure that HCWs are able to absorb the content effectively. The facilitation needs to be consistent, regular and provided by experienced HCWs [38]. Bing-Jonsson et al. [4] also stress that competence development within an NH setting should be practice-based, include authentic examples of interaction [39] and facilitate reflections on one’s own practice [19, 40]. A systematic review of psychosocial interventions in NHs showed that experiential learning, interactive training, supervision and video feedback support HCWs in recognising and evaluating their practice [41]. It has been proposed that further research on psychosocial interventions and communication training should explore how competence development can enhance the quality of psychosocial and person-centred practices [42, 43] and reduce the theory-practice-gap in PCC in NHs [7, 8, 11].

### International caregiver development programme

ICDP is a preventive psychosocial competence-building programme. It originated at the University of Oslo as International Child Development Programme (ICDP), designed for caregivers of children, and has been implemented in approximately 70 countries [44–46]. The child-focused version has been adapted for caregivers of older persons [47, 48] and is currently in use in Sweden, Denmark, Germany, Japan, Colombia and Norway. This is the first empirical research project of ICDP for older persons (see also Holmsen et al., 2025; Holmsen et al., 2023 for publications from the same study).

The aim of ICDP in care for older persons is to help HCWs to view care recipients as unique individuals and to develop sensitivity and confidence in their practice. Key concepts that anchor ICDP’s humanistic values include communication, interaction, empathetic identification, intersubjectivity, PCC, salutogenesis (emphasises the individual’s resources and understanding of how people may define themselves as healthy despite for experiencing stress and health challenges). resilience, positive psychology, self-reflexivity, sensitization and sociocultural learning. ICDP meeting content begins with the prerequisites for being a caregiver, progresses to interactions with older persons as unique individuals, and concludes with ethical, cultural and taboo-related topics relevant to healthcare services for older adults. The programme is designed to be flexible and accessible for individuals from diverse professional, cultural and linguistic backgrounds.

Eight guidelines, grounded in global research on universal psychosocial needs, are central to helping participants articulate tacit knowledge and apply it to psychosocial practice. Competence development arises from identifying the eight ICDP guidelines in participants’ video recordings of their practice, as well as through other activity-based and sensitising educational methods. In this way, ICDP focuses on mastery and builds on the existing strengths of staff. Its content and pedagogical approach aim to foster empathetic identification and ability to understand own and others interactive behaviour in light of thoughts, feelings and needs; mentalisation, spark reflection and support an ongoing reflective process and a more conscious, systematic approach to psychosocial and person-centred practice [18, 47, 49].

The video recordings that are used in ICDP capture interactions between resident and ICDP participants in ordinary everyday situations, such as mealtimes. The ICDP participants are told to avoid depicting sensitive situations of residents, although all employees in nursing homes in Norway are bound by legislation of confidentiality. The ICDP guidelines facilitate the analysis of interactions, with a primary focus on the actions of the employees. All residents who participate in the filming for use in ICDP groups are required to provide informed written consent. The consent may be obtained from peers or guardians if the residents is not able to consent. The consent process adheres to applicable legislation and is stored in the residents’ records. The films are usually recorded by a colleague and should be recorded on a camera that does not have internet coverage.

### The international caregiver development programme facilitator training

In this study, the certification of ICDP facilitators involved six days of theoretical training. The ICDP facilitator candidates were recruited by their leaders, who were encouraged to recruit HCWs with different backgrounds to reflect the diversity of HCWs in NHs. The practical training included leading (organizing and facilitating) ICDP groups, following a minimum of two supervision sessions. This practical component was carried out in pairs and consisted of eight ICDP meetings with 5 to 8 colleagues at the facilitators’ NHs. To become certified as ICDP facilitators, the group leaders submitted a log with a fixed format from each ICDP meeting, in addition to a reflection on their facilitation.

Before leading ICDP groups as part of their training, the facilitator candidates received an ICDP handbook and meeting plans with suggested exercises for each topic. They were also given a bank of ideas for facilitation methods and ICDP content and were encouraged to adapt their facilitation to the context of their group participants.

The five multicultural ICDP groups in this study included 33 HCWs with diverse educational backgrounds, job roles and years of experience, working in both open and screened units of medical or dementia wards. The groups represented 17 different languages. On average, participants attended seven out of the eight ICDP meetings. For more information about the intervention and ICDP participants, see Holmsen et al. [50].

## Methods

### Aim and research question

The aim of this study was to examine adaptations to competence development within PCC in multicultural healthcare teams that support knowledge integration and reflexivity in practice. The research question is: In what ways can group leaders in ICDP facilitate competence development within psychosocial care for employees in nursing homes?

### Design

This article is one of three based on data from the first empirical study examining ICDP in care for older persons [49, 50]. A qualitative design was chosen to explore the group leaders’ in-depth experiences of facilitating ICDP for HCWs in NHs, and to inform future quantitative and qualitative studies [51]. The qualitative approach allows for a more flexible framework and supports innovative methods [51, 52].

### Sample

A prerequisite for inclusion in the study was participation in the ICDP facilitator training for NH employees in one of the largest cities in Norway. The nine participants, from three different NHs, were group leaders working in pairs of two to facilitate the five ICDP groups that began their practice training first. One group leader facilitated two different groups in parallel.

The ICDP facilitator candidates were invited to participate in the study by their leader, in cooperation with the researchers. Participants received oral and written information about the study, emphasizing that participation was voluntary and that they could withdraw at any time without consequences. The nine group leaders consented to take part in two focus group interviews and to participatory observations of their supervision sessions, as well as to share their logs from the group meetings and their written reflections.

The nine group leaders were born in five different countries and included 7 women and 2 men, aged 27 to 59 years. Seven were registered nurses, and two were enrolled nurses. Two of the nurses had additional formal education. Their mother tongues were Somali, Filipino, Romanian, Portuguese and Norwegian. They had worked in nursing homes for between 1.5 and 37 years, with an average of 12 years, and most also had experience from other work.

### Data collection

The data for this study were collected in 2018 and 2019 and consisted of five participatory observation sessions during the facilitators’ practice training, the group leaders’ written logs and reflections from the eight ICDP meetings, and one focus group interview conducted after the training period (see Table 1).

**Table 1:** The data material

**During the eight ICDP group meetings**	1) Fieldnotes from five participatory observations of supervision for ICDP group leaders Focus: Mastery and challenges in facilitating ICDP for health care workers in nursing homes 2) The group leaders’ written logs from the eight ICDP meetings. and 3) The group leaders’ reflections conducting ICDP
**After completing ICDP** **and the facilitator training**	4) One focus group interview Focus: Experiences of facilitating ICDP for HCWs in NHs

The participatory observations of the mandatory supervision sessions were conducted in the three nursing homes by the first author. Supervision with the 9 group leaders was led by an ICDP trainer and consisted of 2 sessions of 75 min each. The first session focused on the status of the groups and the facilitators’ experiences of mastery and challenges in facilitation. The second session involved supervision and dialogue on strategies to prevent or address these challenges. The researcher mainly participated in the second session, taking part in the open discussions about strategies for improving ICDP facilitation. Please see additional file 1 for information about the guide for participatory observations. The fieldnotes contained data from the group leaders’ dialogues about group status, as well as their perceived mastery and challenges in facilitating ICDP. The group leaders had conducted a various number of group meetings by the time they attended the supervision sessions.

One focus group interview, lasting 90 min, took place at VID Specialized University and was conducted by 2 of the authors in collaboration with an ICDP trainer. The aim of the interview was to explore the group leaders’ overall experiences of facilitating the eight ICDP meetings in retrospect. Please see additional file 2 for information about the interview guide for the focus group interview. The interview was transcribed verbatim to preserve the group leaders’ own ways of expressing themselves [53].

The group leaders’ written logs and reflections were part of the ICDP facilitator certification process. The five logs were structured according to the eight ICDP meetings and included descriptions and assessments of planning, content, structure and level of engagement. Please see additional file 3 for information about the template for logs and reflections from the ICDP meetings. The nine reflection notes from each group leader offered reflexive reviews of their experience facilitating ICDP in nursing homes. These logs and reflections were submitted to the ICDP trainers, anonymised and then prepared for analysis by the researchers.

All fieldnotes, logs, reflections and the focus group transcript were stored on an encrypted flash drive, transcribed by the first author and transferred to VID’s research data storage server. Pseudonyms were used to ensure anonymity.

### Analysis

The qualitative analysis was conducted in 2024 and 2025, and was inspired by Brinkmann and Kvale’s [54] description of hermeneutic analysis. The process involved moving back and forth between the whole and parts of the data, using mind maps to identify themes, structuring quotations and assessing whether the structure reflected the data material accurately.

The analysis began with repeated readings of the focus group transcript and fieldnotes in various orders while maintaining an open-minded approach. Mind maps were developed to explore different ways of structuring the data. Over time, an emerging overview suggested that it would be valuable to explore how the group leaders adapted their facilitation to suit the ICDP participants. The analysis of the fieldnotes and focus group interview was then integrated and guided by the following analytical questions: [1] What did the group leaders do to facilitate ICDP for employees in nursing homes [2]? How did they experience facilitating ICDP for employees in nursing homes [3]? How did they adapt ICDP to support HCWs’ learning processes in psychosocial care? And [4] How did these adaptations affect the NH employees?

The mind maps were restructured from focusing on group leaders, ICDP content and learning methods, and ICDP participants, to focusing on the facilitation of atmosphere, content and participant engagement. Before writing up the findings, the material was coded and categories were adjusted using quotations from the group leaders’ logs and reflections on the group meetings.

Study findings were written up and illustrated with selected quotes. The themes and quotes were revised repeatedly throughout the process of preparing the presentation of results. All quotes were translated from Norwegian to English and then back-translated to ensure that their meaning was preserved. Changes to original wording are indicated with square brackets ‘[…]’.

Before finalising the results section, the entire data set was re-read to assess whether any new insights might be drawn. The presentation of findings seeks to reflect how the four different data sources complemented one another in illuminating the facilitation of ICDP from various perspectives. An initial decision to exclude the logs was reconsidered, as they provided insights into group processes not captured by the other materials.

The analysis was carried out in collaboration with all authors. Tentative results, theme revisions and interpretations were discussed during regular analysis seminars with the author team and through a presentation to a research group at VID Specialized University. The presentation of results was also reviewed by the ICDP trainer who had conducted the supervision sessions with the group leaders. She found the results to be recognisable and insightful and had no suggested corrections.

### Ethical considerations

The study was conducted in accordance with the Declaration of Helsinki and received approval from the Norwegian Centre for Research Data (NSD); project number 332,083, in October 2018. According to the Norwegian Centre for Research Data, there was no requirement for approval from the Regional Ethical Committee (REK) for the conduct of this study. Consent forms, coding keys, contracts and other relevant information pertaining to all participants, were securely stored in a locked cabinet at VID Specialized University.

The first author served as both a researcher and the lead for the implementation of ICDP in nursing homes. This dual role raised specific ethical considerations with regards to trustworthiness and authenticity [55], that were sought mitigated by: A written account of the author’s assumptions was recorded before data collection began [56]. The research methodology was designed to promote transparency and allow readers to critically assess how preunderstandings may have influenced the study [54]. The Consolidated Criteria for Reporting Qualitative Research (COREQ) was used to guide the reporting of this study. For further methodological details, see Holmsen [49].

## Results

Please see Table 2 for a comprehensive summary of the results.

**Table 2:** Results

**Creating the right atmosphere** Creating safety for openness Highlighting mastery in practice Helping the ICDP participants to be mentally attuned
**Making ICDP Understandable** Transitioning to a reflexive mode Adapting the language level
**Creating an inclusive and active learning environment** Facilitating collective participation Supporting the groups’ engagement

### Creating the right atmosphere

The group leaders emphasized the importance of establishing a secure atmosphere to encourage openness during the initial meetings. Openness and emotional expression led to mutual support within the ICDP groups. Fieldnotes and the focus group interview indicated that highlighting mastery gradually improved ICDP participants’ self-confidence. This was important for reducing reluctance to participate in role-plays, discussions and video recordings –reluctance that was interpreted as stemming from discomfort, shyness and insecurity about making their practice visible. The fieldnotes also pointed to time pressure and stress from limited resources as the greatest challenge to implementing ICDP in nursing homes. This made it necessary for group leaders to create a calm atmosphere to support participants’ present-moment awareness during the group meetings.

### Creating safety for openness

The fieldnotes and logs suggested that information about ICDP fostered positive expectations, and that establishing a consensus on ‘ground rules’ helped to create predictability and a sense of safety. The group leaders emphasized the importance of adjusting activities to the group’s atmosphere to support a sense of security during ICDP exercises.

*At a supervision that took place in a NH*,* the four group leaders were asked about their groups’ progress. Amanda and Maria described how*,* during their first ICDP meeting*,* participants had felt unsafe before the first exercise. They had told participants that they could share only what they felt comfortable sharing. To help ease any insecurity*,* the group leaders began by sharing their own experiences.* (Participatory observation 1)

Group leaders participating in ICDP exercises on equal footing with the participants were reported to foster a sense of safety, guidance and equality. To support a safe environment, some leaders emphasised that differences in experience, education, practice and cultural background were all valuable and meaningful in the ICDP exercises.

The group leaders also expressed a desire for more activities to help HCWs become aware of the emotions that may arise in interactions with residents.

*In a supervision session with seven group leaders*,* Ellen highlighted that staff need to be able to talk about coping with stressful situations at work. She noted that ICDP could benefit from more dialogue about emotions*,* as this was missing in the staff’s daily work routines (Participatory observation 2).*

Openness around emotions, attitudes, values and preconceptions was seen to encourage support, respect, inclusion and a sense of community. Several group leaders noted that using various strategies to build group cohesion had a positive impact on the working environment and the quality of care provided to residents.

### Highlighting mastery in practice

Supporting the ICDP participants’ confidence involved acknowledging that it is unrealistic to expect HCWs to master every challenge in practice. At the same time, the group leaders emphasised the value of recognising and affirming participants’ achievements – especially through video recordings of their own practice.

*In the minutes from Group Meeting 4*,* Maya and Helene noted: ‘It was clear that those who had created [and presented] video recordings developed confidence from the group’s feedback’* (Log from ICDP meeting 4).

Identifying the eight ICDP guidelines enabled targeted feedback on interaction details, affirming participants’ competence and increasing their confidence and pride in their work. Facilitating a safe environment for sharing video recordings was supported by using small devices like mobile phones or tablets rather than a large projector screen, and this felt more intimate.

Several group leaders preferred to highlight the ICDP participants’ existing competencies rather than rely on instructive lecturing. However, they were careful to distinguish between recognising mastery and offering generic praise for skills that might otherwise be taken for granted.

*After conducting four ICDP meetings*,* Helene and Ellen attended a supervision session. Reflecting on ICDP’s focus on mastery*,* Ellen emphasised that recognition should not mean praising routine aspects of care. Instead*,* she noted*,* feedback should build on the many resources participants already bring to their work.* (Participatory observation 2)

The ICDP participants’ growing self-confidence became evident in two main ways: their increased engagement during discussions in professional NH forums and their willingness to document challenges in practice. Some surprised the group leaders by voluntarily recording difficult interactions and initiating conversations about taboo subjects. Several group leaders wanted to place even more emphasis on building self-confidence and recognising those participants who seemed in particular need of support.

### Helping the ICDP participants to be mentally attuned

Participants often struggled to transition into what group leaders referred to as ‘ICDP mode’ – a reflective state described as being mentally attuned, present in body and mind, and emotionally aware.

*After conducting three ICDP meetings Amanda and Maria participated in a dialogue with two other group leaders about the challenges in facilitating ICDP. Amanda and Maria talked about how the participants had expressed that they were fatigued. The leaders said they had to ‘pull and tug’ and almost reflect for the ICDP participants: ‘It felt as if the room was completely grey when we started’. The two left the meeting feeling discouraged.* (Participatory observation 1)

Because it was often difficult to distinguish between fatigue and a lack of motivation, one notable moment was when a mindfulness exercise received positive feedback – after encouragement and recognition had failed to engage the ICDP participants. In response to signs of fatigue, some group leaders tried introducing physical activity to help participants refocus. All group leaders found that playing calm music or reading poetry helped participants to relax and transition into a reflective mindset.

*In the minutes from group meeting 1*,* Nicole and Angelica’s noted: “We started the meeting by playing music […] We noticed almost immediately that*,* due to the music*,* the participants calmed down and shifted to a reflective mode.”* (Log from ICDP meeting 1).

Several participants brought music and poetry into their departments and expressed interest in incorporating more of these elements into ICDP. As the ICDP required significant time and energy from both leaders and participants, group leaders who conducted weekly meetings suggested that biweekly meetings might help to alleviate time pressure.

### Making the ICDP understandable

The data indicated that ICDP participants relied on group leader support and peer support to transition to a reflected mode to practice. Challenges in becoming reflective were tied to the everyday use of tacit knowledge and the unconscious, intuitive nature of psychosocial care in a busy work setting. Field notes, log and the focus group interview all highlighted how participants’ varying and sometimes limited Norwegian vocabulary required the facilitation to be adapted to participants’ language levels. The ICDP was described as a valuable arena for developing both language and professional vocabulary within a team consisting of people with diverse linguistic and professional backgrounds. The length of the chapter “Transitioning to a reflective mode” is reflecting the data material and the importance of this specific finding.

### Transitioning to a reflective mode

As stated in the focus group interview, the group leaders described the ICDP content as easy to understand, recognizable and close to practice:

Sverre: *Many associated ICDP with person-centred care … there are similarities* (Focus group interview)

After completing the ICDP, several group leaders noted that grasping the content through active participation in group meetings was easier than learning by reading it in a book.

Initially, the group leaders encountered challenges in facilitating the pedagogical methods designed to support the integration of ICDP content into practice. They explained that many ICDP participants began by treating the eight ICDP guidelines as fixed ‘facts’ rather than as tools for reflection. Participants needed support in articulating and reflecting on both verbal and nonverbal communication. It was particularly difficult for some to move from concrete, task-oriented thinking to reflecting on complex interpersonal interactions. Descriptions like ‘We usually do it this way’ made it challenging to elicit the uniqueness and complexity of specific care situations with residents.

Facilitating reflection in the ICDP groups was described to be demanding:

*After Helene and Maya had conducted five*,* and Ellen and Helene had conducted six ICDP meetings*,* they discussed the challenges of facilitating reflection. Helene concluded that close follow-up and the availability of the ICDP trainers had been crucial*,* especially since this was their first time facilitating ICDP.* (Participatory observation 4)

A striking difference in participants’ ability to reflect on practice emerged in two of the logs when two groups from different units were merged for the final ICDP meeting. The two pairs of group leaders wrote in their respective logs:

*Group 1 (anonymized): “The staff working in the sheltered units* [group 1] *have a different approach to ICDP* [than group 2]. *Our group is very theoretical*,* and it takes some time to get into ICDP mode.” (Log from ICDP meeting 8)*.

*Group 2 (anonymized): «We observed big differences in the two groups*[…] *our group is like “born to ICDP*[…] *sheltered unit have another approach to ICDP.» (Log from ICDP meeting 8)*.

In line with this, in a participatory observation session, Helene explained that HCWs in somatic care, who ‘work more with their mind than their heart’, may find it more difficult to shift focus towards psychosocial care (Participatory observations 2).

The data indicated that ICDP participants relied on the group context for support in transitioning to a reflective mode:

*In one of the supervision sessions*,* seven group leaders discussed difficulties in engaging participants in the practice assignment between meetings. After conducting six ICDP meetings*,* Amanda and Maria noted that their group members struggled to reflect on their own but were able to reflect well when in dialogue with the group.* (Participatory observation 2)

Analysis showed that participants gradually developed the ability to connect the professional concepts in the eight ICDP themes to detailed reflections on their own practice. Role-plays, practice examples and especially video recordings proved to be effective pedagogical tools for linking psychosocial care concepts to everyday practice.

*Sverre and Espen noted: “We analysed the video recordings showing the participants’ practice*,* based on the ICDP guidelines we had examined… This was likely a good choice*,* as it made understanding easier.”* (Log from ICDP meeting 5).

Group leaders found it beneficial to clarify and reflect on one ICDP guideline at a time. Reading poetry tailored to each guideline was reported to stimulate reflection on practice.

*Sverre and Espen had conducted their seventh ICDP meeting the day before a supervision session held in a NH with five group leaders present. In discussing the status of their group*,* Sverre noted that the participants had started to value visualising and reflecting on their practice: not just learning how to complete tasks but also thinking about what they were doing and how they were doing it*. (Participatory observation 5)

Several group leaders reported that ICDP participants gained “new glasses” through which they viewed themselves and their work. In one of the two participating nursing home units, the institutional manager observed that staff had become more reflective following their participation in the ICDP:

Ellen: *Following the Resident Safety Visits*,* our manager noticed that staff at [Unit 1]*,* spoke differently about the residents and had a different focus and reflected more on new issues.* [Unit 1] *demonstrated a higher level of reflection compared to* [Unit 2], *which previously had a higher reflection level than* [Unit 1]. [The manager] *believed that ICDP had an important impact*,* as no other changes were introduced besides ICDP.* (Focus group interview)

The data contained numerous examples of group leaders’ observations about participants’ growing use of professional terminology, awareness, reflexivity, understanding, sensitivity and a more systematic approach to psychosocial care.

One group leader described how ICDP participants incorporated psychosocial care into their daily work but still struggled to distinguish among the eight ICDP guidelines and connect them meaningfully to their own practice. Like others, this group leader suggested that the number of ICDP meetings should be adjusted to meet participants’ differing needs.

### Adapting the Language level

Group leaders expressed concern that participants’ varying and often limited Norwegian vocabulary might make it difficult to explain the significance of professional psychosocial concepts. They also noted concerns about participants’ ability to express themselves. However, the introduction of video recordings and role-plays helped participants articulate details in their practical knowledge, supported by input from the group.

Espen: *When we went through the ICDP guidelines… it is very concrete and straightforward… they can recognise what they… are doing… and… become able to articulate it.* (Focus group interview)

Group leaders observed improvements in participants’ use of professional terminology and language throughout the ICDP process. Several noted that they could have spent more time on language development and wanted to include more repetition and summarisation of ICDP content in future groups.

Although the group leaders felt that the ICDP materials were helpful, they also found the language and terminology to be complex – and that they lacked the time and expertise to adapt the meeting plans and materials to participants’ language needs. Some had positive experiences using a simplified glossary tailored for the ICDP. Others emphasised the need for support from nursing home management to allow more time for meeting preparation.

### Creating an inclusive and active learning environment

To create an inclusive and active learning environment, the facilitation of collective participation required the group leaders’ sensitive regulation of the ICDP participants’ high levels of engagement. At the same time, it could be demanding for the group leaders to sustain varying levels of engagement, motivation and enthusiasm among participants and to assess where to draw the line when ‘pushing’ them.

### Facilitating collective participation

Group leaders linked the participants’ high engagement in the ICDP meetings to a lack of time for dialogue in their everyday work. However, this high level of engagement also created challenges in facilitating and managing group dialogues.

*The four present group leaders reflected on challenges*,* when Nicole and Angelica described how participants spoke extensively during their first ICDP meeting. Nicole explained: ‘We lost control… The dialogue was galloping.”* (Participatory observation 1).

The group leaders were concerned that regulating these discussions might lead to feelings of rejection and a decline in engagement. At the same time, they did not view high engagement negatively – particularly when it encouraged participation from others in the group.

To facilitate collective participation, group leaders used strategies such as pairing participants for the dialogues, forming small groups of three conducting role-plays and involving everyone in a round-table dialogue. This established an expectation of collective participation in the dialogues and fostered a sense of community and engagement among the ICDP participants.

*Helene and Maya*,* who had conducted three ICDP meetings before their first supervision*,* described how a previously quiet participant became very vocal during the ICDP dialogues. Helene explained that she believed this shift stemmed from being given the opportunity to express herself and be listened to.* **(**Participatory observation 2)

Group leaders also observed that ICDP participants began to apply these communication skills in other meetings within the nursing home, resulting in a more constructive tone.

A recurring concern was the ‘balancing act’ between using time effectively and avoiding taking too much of participants’ time. Group leaders described challenges in maintaining focus, structuring the meetings and keeping discussions on track. Some expressed a wish to become more conscious and intentional in their role as dialogue facilitators.

### Supporting the groups’ engagement

Group leaders expressed concern that confusion about how to reflect on practice – combined with fatigue from frequent participation in various workplace interventions – might negatively affect participants’ engagement in the ICDP. They shared numerous examples of how they adapted the ICDP to support varying levels of engagement. Several noted that engagement increased when the content felt personally relevant, especially when connected to participants’ direct interactions with residents.

Ellen & Helene noted: *The participant’s video recordings of interactions were highly engaging. Role plays*[…] *engaged them to a lesser degree*[…] [We] *therefore spent a lot of time*[…] [with] *the greatest focus on the films and connected the guidelines to them*. (Log from ICDP meeting 3)

Exercises involving active participation and reflection on cultural aspects or ethical dilemmas were also seen to spark engagement. Group leaders believed that their own enthusiasm for ICDP and pride in the group process had a positive influence. Still, they expressed that facilitating ICDP was demanding, and that they depended on the suggested meeting plans as a framework for conducting the eight sessions.

Engagement with ICDP appeared to increase over time as both leaders and participants better understood its purpose. When participants questioned the point of engaging in quality improvement processes that often lacked long-term follow-up, group leaders responded by involving them in developing a plan to integrate ICDP into existing routines and documentation systems. By the final meetings, several participants even attended ICDP sessions during their time off. Group leaders reported numerous examples of improvements in both the quality of care and the working environment. Two group leaders reflected:

Amanda: *“There are benefits in terms of the working environment* that *will* certainly […] *benefit the residents. I find this interesting*[…]*whether the sick leave*[…] *has decreased and believe that it should be investigated. Because I actually think it has.“*

Nicole: “*Maybe that’s why*[…] *the sick leave in our unit has changed. There has been zero sick leave*[…] *which is wonderful.”* (Focus Group Interview).

## Discussion

This study aimed to examine competence development within psychosocial care and PCC, with a focus on how group leaders facilitated the ICDP for nursing home employees. The group leaders sought to create an atmosphere characterised by calmness, mastery and security to facilitate present-moment awareness and openness about emotions and practice. To become reflexive and develop a language for psychosocial practice, the ICDP participants needed support from the group and facilitative methods that were closely connected to their everyday practice. To foster an active and inclusive learning environment, the group leaders regulated the dialogue and adapted the content of the meetings to participants’ levels of engagement and motivation.

### Reflexivity as a prerequisite for person-centred practice

The results suggest that ICDP may contribute to realising PCC. The content of the ICDP was accessible and comprehensible to HCWs in nursing homes. However, the HCWs – particularly those from somatic units – faced challenges in using the ICDP guidelines and in shifting from task- and fact-focused, concrete thinking to a more reflective approach to practice. This aligns with studies noting that education and nursing home practice are often dominated by somatic and task-oriented care [1, 8, 10]. To realise PCC in practice, HCWs must be supported to shift between task-oriented routines and practices that require reflection [20]. The ability to reflect on one’s own practice is important for assessing individual needs and recognising the complexity of interactions, which are essential for holistic PCC and for fostering ongoing learning processes in person-centred practice [19, 22]. Several studies emphasise the importance of exploring ways to reduce the theory–practice gap in PCC [7] including through practice-oriented and simulation-based pedagogical methods for competence development in person-centred and psychosocial care ( [4, 31, 39, 41, 57]. In the present study, HCWs appeared to find it challenging to reflect on their own. The findings suggest that using the eight ICDP guidelines alongside video recordings of participants’ actual practice can serve as valuable tools for visualising what PCC looks like in real-life settings – and for engaging HCWs in verbalising and reflecting on their practice.

Tashiro et al.’s [19] observation that reflection often emerges from emotional challenges within practice is particularly relevant to ICDP’s sensitising pedagogical approach. To enhance HCWs’ emotional awareness in psychosocial care, ICDP encourages participants to imagine both the residents’ and their own emotions during interactions. The shift from participants’ initial reluctance to openness to being able to reflect on their most challenging interactions – aligns with Tashiro et al.’s [19] emphasis on the role of emotionally challenging practice. ICDP’s focus on safety, mastery and present-moment awareness appears to support the development of such reflective capacity.

The findings also indicate that group-based interventions are essential to developing HCWs’ reflectiveness. In line with Tashiro et al. [19], this study shows that reflexivity and competence development in PCC arise through interaction with others. Beyond reflecting in relation to residents, the findings highlight that nursing home staff also depend on mutual support from colleagues within the group to become reflective.

### Adaption of psychosocial interventions to a nursing home context

In line with recommendations from other research [14, 38], this study highlights the importance of adapting interventions to the context in order to foster competence development in psychosocial care and PCC. It suggests that integrating ICDP within multicultural healthcare teams in nursing homes requires adjustments related to openness about emotions and practice, recognition and safety in one’s own practice, present-moment awareness, challenges with transitioning to a reflective view of practice, as well as the participants’ language skills, engagement and motivation. These adjustments may be crucial for integrating knowledge of PCC in contexts characterised by limited resources, a culture dominated by task-focused somatic care, low professional confidence and challenges related to language competence, diversity and discrimination [10, 32–37]. Facilitating psychological safety for HCWs to be open about emotions and practice is important for the development of interpersonal and intrapersonal skills, which are considered fundamental for providing psychosocial care and PCC [13, 26–28].

Consistent with recommendations from other studies on competence development [4, 14, 38, 41, 58] the findings indicate that a group-based, flexible intervention – grounded in sensitising and simulation-based methods, and inclusive of emotional support and motivation – is appropriate for the nursing home setting. ICDP’s flexibility allows for adjustments that may help HCWs to grasp and integrate new knowledge and skills essential for transitioning to person-centred practice [31]. This is relevant in efforts to reduce the theory–practice gap in PCC. However, the ICDP group leaders expressed desire to make further adjustments suggests that the implementation could have been even better tailored to multicultural teams in nursing homes. In this study, while the ICDP content related to psychosocial care remained consistent, the educational and simulation-based methods were adapted to the local context. The study thus indicates that what matters most for participant engagement is adapting the facilitation to each unit and nursing home context.

### Flexibility and sustainability

This study tangent important aspects with regards to the global workforce issue [59]. Research points to the challenges that can arise when an intervention is originally developed for a different context. ICDP was originally developed for children, based on the idea that caregivers’ resources are the starting point for development, and that psychosocial needs remain the same from birth to old age. The present study indicate that ICDP can be adapted to a nursing home context – however, group leaders reported that facilitating ICDP while tailoring it to participants required significant effort. Moreover, Manson et al. [38] argue that PCC interventions should be facilitated by experienced HCWs. While the group leaders in this study were experienced with regards to care for older persons, they were novices in facilitating ICDP. The group leaders emphasised the importance of having access to structured meeting plans, alongside the flexibility to adapt those plans to the nursing home context. They also underlined the need for support from management and sufficient time to prepare before and after each ICDP meeting. Despite being first-time facilitators, the group leaders demonstrated sound facilitation skills and sensitivity to the adaptations needed for multicultural nursing home teams. They expressed both enthusiasm for ICDP and pride in their role as facilitators.

In line with Holmsen et al. [49], the findings suggest that HCWs grasp and integrate ICDP at different paces, regardless of how facilitation is adapted. The study indicates that ICDP is most easily understood by HCWs working in long-term dementia units. One of the group leaders from a somatic unit argued for extending the group meetings to enable the HCWs to integrate reflexivity on their practice. This raises the question of whether ICDP is equally suitable for all types of nursing home units. Considering research that highlights the importance of quality in PCC [60] and the need to focus on both somatic and psychosocial care in NH [20, 21], it may be especially important to integrate ICDP in somatic units in NHs.

In line with recommendations emphasising HCW motivation in PCC interventions [14, 38], the ICDP participants in this study appeared to become increasingly motivated over time. Their motivation may have stemmed from improved ability to reflect on practice, enhanced professional confidence and an increased sense of well-being – all of which may have contributed to improved quality of care, a better working environment and reduced sick leave [61]. At the same time, ensuring that ICDP can be integrated into existing structures after the eight meetings is likely important for maintaining participants’ motivation. Further research is needed on the sustainability of ICDP in nursing homes. Additionally, the findings related to reduced sick leave would be valuable to investigate in a larger quantitative study.

### Methodological considerations

The triangulation of different data collection methods and a comprehensive data set represent strengths of this study [51]. However, the results should be interpreted in light of several limitations. The findings from participatory observations, logs and self-reflections may have been influenced by the fact that the participating group leaders were seeking approval for their ICDP facilitator certification to become facilitators in ICDP to be approved. Therefore, a focus group interview was conducted after their certification process had concluded. The results from the focus group interview were found to be largely consistent with the rest of the data material. To address potential threats to the study’s validity, the research team was composed of members with diverse methodological and academic backgrounds, who collaborated closely throughout the study. To minimise potential bias, we presented the study findings in various forums and received feedback that included reflections on the first author’s dual role and contributions.

Study findings are based on the facilitation of ICDP among HCWs in a nursing home setting and may therefore be most relevant for similar interventions within nursing home healthcare teams. At the same time, the transferability of this qualitative study should be viewed as constrained by and dependent on factors such as the specific mandate of the healthcare services, culture of care, structural frameworks, legal context and leadership dynamics. Additional quantitative research is necessary to confirm the effectiveness in relation to the sustainability of ICDP and the reduction of sick leave.

## Conclusions

Study findings suggest that interventions for psychosocial competence development among multicultural nursing home employees require adjustments related to HCWs’ needs for security, a sense of mastery, present-moment awareness, opportunities for reflection in practice, linguistic level, commitment and motivation. These adjustments may be crucial for reducing the theory–practice gap in person-centred practice, by supporting HCWs in transitioning between task-focused work and reflective practice. ICDP appears flexible and adaptable to multicultural healthcare teams and seems appropriate for supporting HCWs in integrating knowledge, reflexivity and sensitivity into person-centred practice. This is important for improving the quality of care for vulnerable nursing home residents. However, interventions within PCC may be challenging to facilitate, particularly when it comes to enabling HCWs to shift towards reflective practice. Adaptable interventions also require sufficient supervision for group leaders and adequate time for preparation. This study points to aspects with regards to the global workforce issue. Further research is needed on multicultural healthcare teams’ transition to reflectiveness, the impact of tailoring psychosocial interventions to the nursing home context, and the sustainability of ICDP. In addition, studies examining ICDP’s effect on professional confidence and sick leave are warranted.

## Supplementary Information

Below is the link to the electronic supplementary material.

Supplementary Material 1

Supplementary Material 2

Supplementary Material 3

## Data Availability

The datasets generated and analysed as a part of the current study are not publicly available because the data are qualitative in nature and could potentially result in identification of the participants. Anonymized transcripts are available from the corresponding author upon reasonable request.

## Abbreviations

HCW: Healthcare worker
ICDP: International Caregiver Development Programme
NH: Nursing home
PCC: Person-centred care
