# Supplementary material for: Adapting competence development to multicultural healthcare teams: a qualitative study of the International Caregiver Development Programme (ICDP) in nursing homes
Source: BMC Nurs. 2026 Jan 9;25:74. doi: 10.1186/s12912-026-04299-3 (PMC12829293; doi:10.1186/s12912-026-04299-3)
Supplement: Supplementary file 1 — Supplementary Material 1 [file 12912_2026_4299_MOESM1_ESM.pdf]

Participatory observations

Context: *guidance with group leaders in ICDP*

Participants: *the group leaders in ICDP have 2-3 mandatory tutorials during the group process*

Location: *In one of the nursing homes where ICDP is implemented or in group rooms at VID University College of Applied Sciences*

Observer: *Line Constance Holmsen*

Observer's participation: *primarily wants to have an observer role. May be asked to comment on or supplement the dialogue between the group leaders with things I have observed during the group meetings in ICDP.*

Participatory observations of supervision with the group leaders;

Focus on theme:

- How many group meetings have the different group leader pairs conducted?
- Status in the various groups
- coping and challenges in the group leader role,
- what the group leaders said they did and could do to facilitate the participants through the various exercises in ICDP and how the group participants reacted to it
- What experiences do the group leaders have with facilitating ICDP in general?
- What engages group participants more and less?
- group process

Focus on observation of the dialogue between the group leaders:

- Write keywords about:
- Who is in dialogue with whom about what?
- The process of the dialogues
- Tone of voice, facial expressions; non-verbal communication

Write down everything I get from the dialogues during the tutorial.

Immediately after the tutorial:

- Write down your own impressions and reflections about the guidance and dialogue between the group leaders
- Writing down experiences I experienced as central

Print/transcribe field notes as quickly as possible after observations.
