# Supplementary material for: Adapting competence development to multicultural healthcare teams: a qualitative study of the International Caregiver Development Programme (ICDP) in nursing homes
Source: BMC Nurs. 2026 Jan 9;25:74. doi: 10.1186/s12912-026-04299-3 (PMC12829293; doi:10.1186/s12912-026-04299-3)
Supplement: Supplementary file 2 — Supplementary Material 2 [file 12912_2026_4299_MOESM2_ESM.pdf]

Focus group interview with group leaders in ICDP (International Caregiver Development Program) in nursing homes

Target group: nursing home employees who have participated in supervisor training in ICDP

Number of participants in the focus group: 9

Purpose: Seek to gain knowledge about the group leaders' experiences with the group leader role, the implementation of ICDP and the relevance of the intervention for employees in elderly care.

Time: After completion of the internship period, as well as submission of log, reflection and theoretical requirements.

*The focus group interview is **conducted with two moderators.** One of the moderators controls the floor, the other observes, asks follow-up questions and makes sure that everyone has a chance to speak.*

***Bring** a dictaphone and writing paper – write down key words during the dialogues.*

Semi-structured qualitative interview

Interview guide

When you think back to the supervisor training in ICDP:

What experiences and thoughts do you have about group leader training in ICDP?

- The content
- Pedagogy/methodology
- The sensitization exercises
- The implementation principles and sensitization principles
- Confidence in participating in group leader training and ICDP groups for employees
- The importance of the researcher's presence in the group meetings
- Experiences with what has been motivating and less motivating in ICDP
- The requirements for certification as group leaders in ICDP
-

What experiences do you have with the implementation of the ICDP groups?

What was challenging?

What did you do well?

What was engaging?

What happened to the group participants?

Group processes

- Thoughts on group process in general

- In case of absence

Notice whether the group participants changed in any way during the group process (both in and outside of the group meetings: in practice or collaboration, in meetings, language)

Thoughts on ICDP's sustainability:

Do you think the ICDP work will continue at your institution?

Thoughts on the relevance of ICDP for nursing home employees:

ICDP in relation to other interventions.

Do you think you would recommend ICDP to other nursing homes?

Before we change the topic in the interview or if something is unclear:

Summarize and check if we have understood them correctly:

Do I understand you correctly if I have understood that you think that… .

Could it be that … .
