# Supplementary material for: Adapting competence development to multicultural healthcare teams: a qualitative study of the International Caregiver Development Programme (ICDP) in nursing homes
Source: BMC Nurs. 2026 Jan 9;25:74. doi: 10.1186/s12912-026-04299-3 (PMC12829293; doi:10.1186/s12912-026-04299-3)
Supplement: Supplementary file 3 — Supplementary Material 3 [file 12912_2026_4299_MOESM3_ESM.pdf]

TEMPLATE FOR GROUP LEADERS' LOG AND REFLECTIONS FROM CONDUCITON OF ICDP IN CARE FOR OLDER PERSONS

Background information: ....

Name of supervisors: ....

Number of recruits: ....

Number of conducted meetings: ....

1. Recruitment to the groups
   • How did you recruit and inform?
   • What worked well?
   • What were the challenges, and how were they resolved?
   • What was the gender distribution in the group?
   • Which professional groups were involved and how many of each were there?

2. How did the composition of the group work? (may be elaborated in the log from the meetings)

- How did it work with the number of participants in the group?
- How did gender distribution work? Any special challenges?

3. Where were the meetings held, and how did it work?

4. Who was in the department while the participants were in the group? Did this work satisfactorily?

MEETING LOG (here you write a log from each meeting)

Meeting number:...

Date:..

Supervisors:...

Number of participants: ....

• What was the plan for the meeting? Was the meeting carried out according to plan? If not, what was the reason for that?

• How did the participants react to the different points/topics in the daily plan?

• What engaged them the most? What engaged them less? What made it happen?

• Was there something that was not understood, or something that was disliked?

• What sensitization methods did you use, and how did they work?

• How was the homework received? Did everyone complete the homework? If not, what was the reason for that?

• How active/engaged was the group? Was everyone active? Were any passive? Is it necessary to make any changes at the next meeting so that everyone can have their say?

• Do you think this was a successful, average or unsuccessful meeting? Why?

• Feel free to add stories, tales and examples from the meeting!

• If someone was absent - what was the reason for that?

Summary after completion of all meetings: Here you write a summary of your assessments of how the group and group guidance worked:

• How do you think the group functioned?

• How did the participants evaluate the group?

• How do you think you fulfilled your role as a supervisor?

• How did you collaborate with your partner?

• What do you think you have learned through this process?

• Is there anything you think could have been done differently and that you would change the next time you have an ICDP group?

Adjusted to ICDP in elderly care by Line Constance Holmsen, Oslo 2018
